# Dispersal syndromes and the use of life-histories to predict dispersal

**DOI:** 10.1111/eva.12049

**Published:** 2013-02-11

**Authors:** Virginie M Stevens, Audrey Trochet, Simon Blanchet, Sylvain Moulherat, Jean Clobert, Michel Baguette

**Affiliations:** 1 CNRS Station d'Ecologie Expérimentale de Moulis, USR 2936, route du CNRS 09200Moulis, France; 2 CNRS: Evolution et Diversité Biologique, U.M.R 5174Toulouse Cedex 4, France; 3 Institut Systématique, Evolution, Biodiversité, UMR 7205, MNHN75005, Paris, France

**Keywords:** butterflies, dispersal inference, dispersal distance, ecological niche, gene flow, life-history traits, Rhopalocera

## Abstract

Due to its impact on local adaptation, population functioning or range shifts, dispersal is considered a central process for population persistence and species evolution. However, measuring dispersal is complicated, which justifies the use of dispersal proxies. Although appealing, and despite its general relationship with dispersal, body size has however proven unsatisfactory as a dispersal proxy. Our hypothesis here is that, given the existence of dispersal syndromes, suites of life-history traits may be alternative, more appropriate proxies for dispersal. We tested this idea by using butterflies as a model system. We demonstrate that different elements of the dispersal process (i.e., individual movement rates, distances, and gene flow) are correlated with different suites of life-history traits: these various elements of dispersal form separate syndromes and must be considered real axes of a species' niche. We then showed that these syndromes allowed accurate predictions of dispersal. The use of life-history traits improved the precision of the inferences made from wing size alone by up to five times. Such trait-based predictions thus provided reliable dispersal inferences that can feed simulation models aiming at investigating the dynamics and evolution of butterfly populations, and possibly of other organisms, under environmental changes, to help their conservation.

## Introduction

The response of biodiversity to global environmental changes is a subtle blend of three ingredients: tolerate the new conditions or adapt, disperse to escape, or decline locally. Dispersal is key in all these ingredients as the movement of individuals that induces gene flow has a considerable role in evolutionary ecology (Ronce 2007; Clobert et al. 2009, 2012), for instance, on the evolution of local adaptations (Doebeli and Dieckmann 2003), and it is also central to the spatial dynamics of populations and metapopulations (Hanski 1998, 1999a). If we are to accurately predict, for instance, the distribution shifts or the potential for evolutionary adaptations under climate change, or the spatial functioning of populations in fragmented landscapes, we need accurate information on dispersal (Berg et al. 2010). However, measuring dispersal is challenging as it is unpredictable in space and time (Nathan 2001), and recording movements among local populations is labor intensive and is usually biased by sampling scale limitations (e.g., Schneider 2003; Franzen and Nilsson 2007).

An appealing solution to overcome this difficulty is to infer dispersal ability for populations or species of interest rather than to measure it directly. One option for making such inferences is to identify general patterns in the organization of dispersal ability across individuals, populations or species, and then to search for a trait—or a suite of traits—that parallels these patterns, which can then be used as a dispersal proxy. Body size was the first candidate in this quest, as it may relate to dispersal either directly because locomotion is scaled to body size, or indirectly because dispersal has causal relationships with other size-dependent traits or processes (Bowman et al. 2002; Clobert et al. 2004). As expected, body size and body shape co-vary with movement rate and dispersal distances in several taxa (moths: Beck and Kitching 2007; birds: Dawideit et al. 2009; plants: Thomson et al. 2010; butterflies: Turlure et al. 2010; Sekar 2012; Stevens et al. 2012). However, the power of the predictions that could be obtained from this co-variation is low since this relationship is rather noisy, and therefore casts doubt about its use to predict dispersal (Dawideit et al. 2009; Sekar 2012; Stevens et al. 2012). In line with this, Baguette et al. (2000) showed that the difference in dispersal rates of three butterfly species over a common network of habitat patches could not have been predicted from differences in their body sizes. Nevertheless, wing size is still frequently used as a direct proxy for butterfly dispersal ability (e.g., Fric et al. 2006; Ockinger et al. 2010).

We believe that life history may offer a better alternative to infer dispersal. Indeed, dispersal is tightly woven into an organisms' life history, encapsulated in syndromes associating different life-history traits both at the within- and at the between-species level (e.g., Li and Margolies 1993; Fjerdingstad et al. 2007; Ronce and Clobert 2012; Stevens et al. 2012). The co-evolution of dispersal and, for instance, those traits that promote a fast turnover of individuals within populations, which results in a so-called dispersal syndrome, offers the opportunity to predict dispersal from the value taken by other, better informed traits. Here, we will examine if life-histories could be suitable proxies to predict the dispersal ability of butterflies, either alone or in combination with body size.

To uncover the syndrome of life history associated with dispersal ability and then to measure the quality of the dispersal prediction based on these syndromes, we used dispersal and life-history data previously published for European butterflies. There are several ways of measuring butterfly dispersal, all revealing different elements of the process (Stevens et al. 2010b). Here, we describe dispersal using four different measurements, pertaining either to the rate and distance of individual movements (measured in the field) or to gene flow among local populations (assessed by population genetics). We considered these four different dispersal measurements sequentially, and modeled their relationships with 18 candidate traits (17 life-history traits and wing size) to highlight the syndromes of traits associated with the corresponding elements of dispersal. Then, we retained the combination of traits that gave the best predictive value, and we quantified (by cross-validation) its ability to predict dispersal. In this quantification, we took the inferences obtained from wing size alone as the reference, since wing size was regularly used as a dispersal proxy for butterflies, and we know that its predictive power is low. Finally, we applied the selected predictive model to more than 100 butterfly species for which dispersal was not measured to date, and we explored the general characteristics of dispersal within this group.

## Materials and methods

### Dispersal data

Butterfly dispersal has been assessed by a variety of methods reviewed in Stevens et al. (2010b), and reliable data were available for 50 NW-European species (of 142). The most popular methods include mark-release-recapture (MRR) and inferences from population genetic structure using allozymes. We used the same dispersal data as in Stevens et al. (2010b), here restricted for the sake of statistical power to those measurements available for > 15 species. This filtering retained four measurements of dispersal, detailed in Table 1: three were directly related to inter-patch movements assessed in MRR surveys and the fourth was the gene flow over space inferred by genetic methods using allozymes. Although some allozymes might be under selection in some populations, these loci were discarded before the calculation of *F*_ST_, as explained in Stevens et al. (2010b).

**Table 1:** The four dispersal measurements available in European butterflies used in this study

Dispersal element	Description of the measurement	Transfo.	*N*
Mean dispersal distance	Mean dispersal distance (km) from a of a negative exponential function of the form P(D) = e^−*α* × D^ with D = distance (km), fitted to dispersal kernel (density probability of dispersal distances) obtained from mark-release-recapture (MRR) surveys. Mean dispersal distance (x) = 1/*α*.	x′ = ln(x)	29
Frequency of long-distance dispersal	Probability of >5 km dispersal movements, estimated from a inverse power function of the form P(D) = a × D^−b^ with D=distance (km), fitted to dispersal kernel (density probability of dispersal distances) obtained from mark-release-recapture (MRR).	x′ = log(x)	28
Dispersal propensity	Propensity to leave a patch, estimated from the proportion of recaptures of marked individuals that occurred in patch of initial capture (residents) in MRR surveys. Dispersal propensity is [1−proportion of residents], and is averaged over patches of different size.	x′ = −√x	25
Gene flow	Dispersal ability estimated from gene flow across landscapes, as given by the analysis of allozymes spatial redistribution. Corresponds to [1−*F*_ST_]. *F*_ST_ quantifies the genetic structuring of populations, and hence is inversely related to gene flow. Loci under selection were removed from the calculation.	x′ = 1−√x	26

Transfo. is the function ensuring data normality, and *N* is the number of European butterfly species for which the measure is given in Stevens et al. (2010b).

Each dispersal measurement was available for 25 to 29 species, for a total of 47 species (11 species have all four measurements, and 15 have only one).

### Life history and morphology

Butterfly life-histories were described by 17 traits pertaining to demography, specialization, and behavior (detailed in Table 2), with species values reported by Bink (1992) and Lafranchis (2000). Ten traits described species demography: the fecundity, the adult lifetime (set to 60 days for species with adult overwintering), the voltinism (the number of generations per year), the larval growth rate (averaged over successive generations), the ripe egg load at emergence, the ovigeny index (proportion of eggs already matured at female emergence), the duration of female maturation, the overwintering stage, the flexibility of the life cycle, and the length of the flight period. Four traits described ecological specialization of a species: the thermal tolerance of adults, their habitat range, the dietary niche breadth of larvae, and the strength of a mutual association with ants (myrmecophily). Three behavioral traits were analyzed. For females, we considered the precision in the choice of the laying site (female precision), and the laying strategy that separates single-egg layers from those species that lay batches of ≥2 eggs. For males, we retained the strategy of mate location.

**Table 2:** Life-history traits used to predict butterfly's dispersal with generalized linear models. All traits are available for 142 butterfly species, except the laying strategy that is available for 137

Trait	Trait description
Fecundity	Mean number of eggs laid by females of the species (9 categories).
Adult lifetime	Mean duration (days) of the adult stage. Upper limit set at 60 days for species that overwinter as adults: ranges 5–60 days.
Voltinism	Annual number of generations, from 0.5 (biannual species) to 3 generation/year.
Larval growth rate	Duration (days) of the feeding period for larvae (i.e., without diapause), averaged over successive generations of a year; ranges 16–186 days.
Ripe egg load	Number of mature eggs in female's abdomen at emergence (9 levels).
Ovigeny index	Proportion of full-grown eggs at emergence (ranges 0–1).
Female maturation	Time (days) between female emergence and its first laying: 8 levels, from 1 (1–2 days) to 8 (laying starts after several weeks of diapause).
Overwintering stage	Stage at which the species usually overwinters. 8 categories: from 0 (egg) to 6 (adult), and an additional category for species without overwintering (warm regions).
Flexibility of life cycle	Separates on the one hand species with inflexible life cycle and on the other hand species with prolonged, shortened, or repeated diapause, with facultative estivation, or with staggering of emergences, all considered ‘flexible species’.
Flight period	Length (in weeks) of flight period (averaged over successive generations where relevant); ranges 3–32 weeks. Results from the interplay between adult lifetime and the synchronization of adult emergences, as shown by a low but significant correlation with lifetime (correlation = 0.34, *P* < 0.001: Stevens et al. 2012).
Thermal tolerance	Degree of adult tolerance to temperature extremes and temperature variation (9 levels).
Adult habitat range	Number of different ecosystems in which adults of the species are usually found (ranges 1–7).
Larval dietary breadth	Number of different host plants caterpillars of the species accept: 4 levels: 1 = plants of one species, 2 = plants of one genus, 3 = plants of several genus of the same family, 4 = plants or several families.
Myrmecophily	Degree of association with ants, from 0 (no association at all) to 9 (obligate, long association).
Female precision	Female precision in egg-laying, 9 levels: from 1 (the female lay where it lands, or even flying) to 9: the female choose the exact position (plant species, plant tissue, height, and orientation) before laying each egg or batch of eggs.
Laying strategy	Female egg-laying strategy: segregates single-egg layers from those species that lay batches of ≥ 2 eggs.
Mate location	Seven levels in the strategy of males mate location, from 1 = sit-and-wait strategy to 7 = strong lek forming, through 3 = patrolling and 5 = territoriality, and intermediates.

Wing size, here summarized by wing length, was used as the 18^th^ species trait. We used the values reported by Bink (1992), who provided average wing size over sexes and generations in cases where these were polymorphic. Wing size was on average 11–37.5 mm for NW-European butterfly species. Wing size was log-transformed before analyses, given that allometric relationships are usually power shaped (Peters 1983).

### Detection of dispersal syndromes

Our aim was to model the various elements of dispersal in butterflies from their life-history traits, while controlling (if necessary) for their wing size. To that purpose, we built models based on the relationships between dispersal measurements and a selection made among the traits presented in Table 2: 10 demographic traits, 4 ecological traits, 3 behavioral traits, and wing size. Wing size was kept in these models as previous studies showed that dispersal is partly dependent on wing size in butterflies (Sekar 2012; Stevens et al. 2012).

In the preliminary step, we analyzed the shape of the relationships between each of the four dispersal measurements and each of 16 species traits (all but binomial traits). In particular, we inspected if there was some evidence for non-linear relationships (i.e., U-shape or inverted U-shape relationships) that should be modeled using polynomial terms. We found evidence of significant quadratic relationships in 7 cases (of 64), and the quadratic term was marginally significant (*P* < 0.1) in 6 other cases (see Table A1): in all these cases, we modeled the effect of the corresponding trait with a second degree polynomial; otherwise, only simple-term (i.e., linear) effects were modeled.

We modeled the relationship between dispersal and life-history traits by generalized linear models (GLM). For each dispersal measurement, the model selection started with a full model with the effect of all 18 traits; in all cases however this model would be saturated. To select a single simpler model*,* we ran all simpler GLM derived from this full model, with a maximum number of parameters set at 8, to avoid saturation. We compared these simpler models via their Akaike Information Criterion corrected for small sample size (AICc: Anderson et al. 1994) using the *dredge R*-function (Barton 2011). Second, to identify possible interactions between traits, we built models in which we incorporated the variables retained in the top-ranked models of the first step of selection (within 2 points of AIC), this time incorporating all first-order interactions. Again, we ran and compared, via their AIC, all simpler models derived from this model, again with the maximum number of parameters set at 8. The model finally retained was chosen from the models with the lowest AIC obtained in this second step of selection (i.e., within 2 points of AIC): we retained only the model with the highest *R*² as it captured most of the deviance and hence would be better at predicting the dispersal measurement, which was our goal.

In multi-species comparative studies, it may be important to account for the interdependency of species that arose through common-ancestry. However, some traits (or associations among traits) may be not related to their phylogenetic history (e.g., Gittleman et al. 1996), in which case the application of phylogenetic comparative methods may be unnecessary, and even may incur errors (Martins 2000). To verify that this was the case here, we performed a preliminary analysis, exactly as described for GLM, but in which dispersal was modeled by phylogenetic generalized least squares method (PGLS, instead of GLM), where the phylogenetic relationships (taken from Cizek et al. 2006) among species was taken into account. In these PGLS, we fitted lambda (the parameter that scales the phylogenetic constraint) by maximum likelihood and verified that its value was negligible (not different from zero). This was the case for all models, as such we do not show these PGLS here, but instead show only GLM, where species are considered independent data points.

### Quality assessment of the predictions

After selecting a model for each of the four dispersal measurements, which evidenced the syndrome(s) of life-history traits associated with the corresponding dispersal elements, we assessed their ability to adequately predict the dispersal ability of species. As quality is a matter of comparison, we took the inferences made from wing size only (i.e., a GLM where the only explanatory variable was wing size) as the reference for this comparison.

The quality of the inferences was measured by cross-validation. We used a 75–25% random partitioning of the data set: 75% of species (i.e., 19–22 species) were the training partition used to parameterize the model (either with the model based on dispersal syndromes, or with the model with wing size only), which was applied to predict dispersal of the remaining species (i.e., 6–7 species in the test partition). One hundred independent random partitions allowed the estimation of standard errors in the predictions.

The performance of each model in predicting dispersal was assessed by comparing observations of dispersal and model predictions. The first measure was the slope of the regression of observations on (mean) predictions, which ideally should tend to +1, and the second measure of performance was the mean absolute difference between observed and predicted values of dispersal. For the mean dispersal distance and the probability of long-distance dispersal, this difference was divided by the corresponding observed value, to account for probable scale dependency in imprecision. We ran 20 independent cross-validations to obtain standard errors of these measures of performance for each model.

The relative performance of the inferences obtained from syndromes of life history rather than from wing size only information was given by the ratio of the mean absolute difference between prediction and observation obtained with both methods, and by the difference between the slopes of observed versus predicted regressions obtained with both methods. The statistical significance of these differences was determined using GLM, with the performance as the response, and the model type as the independent variable.

Finally, to ascertain the relative importance of each variable for the prediction, we partitioned the *R*² of each model among the retained dependent variables, by averaging the increase in *R*² due to each variable over all possible orders of the regressors (see Lindeman et al. 1980). For each term retained for the predictions, we also verified its presence in other alternative models of similar fit (i.e., within 2 points of AIC in the model selection), but that were not used for prediction.

### Dispersal ability of butterflies

We used the four models selected (one per dispersal measurement) to predict the corresponding dispersal element for the 142 butterfly species of N-W Europe. Predictions might be erroneous in cases where the shape of the dispersal/trait relationship remains unknown for a range of trait values. We checked the range of values used to parameterize the model (i.e., in species with measured dispersal) to see if the effect of a given trait was or was not evidenced on a truncated range of trait values. If yes, we restricted our predictions accordingly to the set of species with comparable trait values (see Figs A1, A2 in the online appendix (Data S2)).

## Results

### Dispersal syndromes

The dispersal ability of butterflies tightly correlated with their life-history traits, a pattern that was independent of wing size for three dispersal elements: the mean dispersal distance, the dispersal propensity and the gene flow (Table 3). Wing size was only retained to predict the frequency of long-distance dispersal (Table 3). However, even in this case, the model where life history was incorporated explained the variation in dispersal ability better than did using wing size only. Wing size was thus at best of medium importance in the models with life-history traits (Table 3).

**Table 3:** Linear models used to predict the dispersal of butterflies. Four dispersal measurements were modeled from their relationships with a variety of traits (body size, demography, behaviors and ecological specialization were proposed as independent variables). See text for the procedure of model selection. The lower part of the table shows models with wing size as the only regressor, taken for comparison in this study

Response	GLM selected when 17 life-history traits and wing size were proposed	Contribution to *R*^2^	Estimate	*F*	df	*P*	Adj.*R*^2^
Mean dispersal distance	Intercept	−3.805	−3.805	17.03	11–17	<0.0001	0.863
	Larval growth rate	0.276	−0.032***				
	Adult habitat range 1	0.068	−0.372*				
	Adult habitat range 2	0.092	0.125 (ns)				
	Ovigeny index 1	0.085	−3.187***				
	Ovigeny index 2	0.071	3.249***				
	Mate location	0.104	0.444*				
	Ripe egg load	0.062	0.190***				
	Adult lifetime	0.056	0.053**				
	Mate location × larval dietary breadth	0.043	−0.380***				
	Larval growth rate × mate location	0.032	0.006*				
	Larval dietary breadth	0.028	1.466***				
Frequency of long-distance dispersal	intercept		−3.214	21.45	8–19	<0.0001	0.858
	Length of flight period 1	0.058	−1.273**				
	Length of flight period 2	0.335	−1.906***				
	Log (wing size)	0.148	0.846**				
	Voltinism × adult habitat range	0.142	0.291*				
	Voltinism	0.126	−0.779 (ns)				
	Adult habitat range	0.046	−0.465 (ns)				
	Ovigeny index	0.024	0.328*				
	Larval dietary breadth	0.021	−0.151 (ns)				
Dispersal propensity	Intercept		−0.586	16.79	9–15	<0.001	0.856
	Thermal tolerance, 1	0.037	−0.029				
	Thermal tolerance, 2	0.290	−0.446***				
	Overwintering stage	0.201	−0.122***				
	Myrmecophily	0.164	−0.031***				
	Ripe egg load	0.096	0.036***				
	Female precision	0.039	−0.006***				
	Ovigeny	0.029	0.286*				
	Ovigeny × ripe egg load	0.035	−0.047**				
	Ovigeny × female precision	0.018	−0.040*				
Gene flow	Intercept		0.515	10.09	6–19	<0.0001	0.775
	Fecundity	0.245	−0.004**				
	Female maturation	0.173	0.037***				
	Voltinism	0.122	0.120 (ns)				
	Ripe egg load	0.080	−0.022 (ns)				
	Fecundity × ripe egg load	0.079	0.014***				
	Voltinism × ripe egg load	0.076	−0.023**				
GLM with wing size only							
Mean dispersal distance	Intercept	–	−6.501	11.4	1–27	0.002	0.270
	Log (wing size)		1.529**				
Frequency of long-distance dispersal	Intercept	–	−6.805	9.86	1–26	0.005	0.247
	Log (wing size)		1.571**				
Dispersal propensity	Intercept	–	−1.708	9.87	1–23	0.005	0.270
	Log (wing size)		0.305 **				
Gene flow	Intercept	–	0.753	0.14	1–24	0.720	−0.036
	Log (wing size)		0.001 (ns)				

***P < 0.001; **0.001 > *P*> 0.01; *0.01 > *P* > 0.05; ns: *P* > 0.1.

Contribution to *R*^2^ after the method of Lindeman *et al*. (1980)

A distinct syndrome of life-history traits was associated with each of the four dispersal elements. Each model built here used up to seven different traits pertaining to demography, behavior, and ecological specialization: models are detailed in Table 3 and the trait effects are illustrated in Figs A3–A6 of the online appendix (Data S2). Although we retained a single model for each element, the terms of the model selected were generally also found in most of the other top-ranked concurrent models (see Table A2 of the appendix (Data S2)). Wing size intervened only to predict the frequency of long-distance dispersal, together with adult habitat range and ovigeny voltinism, length of flight period and larval dietary breadth. The mean dispersal distance was best predicted from a combination of seven traits pertaining to demography, specialization, and behavior. Almost completely different suites of traits were retained to explain the variation in the two other dispersal elements. Dispersal propensity was related to thermal tolerance, overwintering stage, myrmecophily, ripe egg load, female precision, and ovigeny. Four traits were needed to explain the variation in gene flow among species: the voltinism and three female traits (the fecundity, the ripe egg load, and the female maturation).

### Quality of predictive models

**Figure 1:**
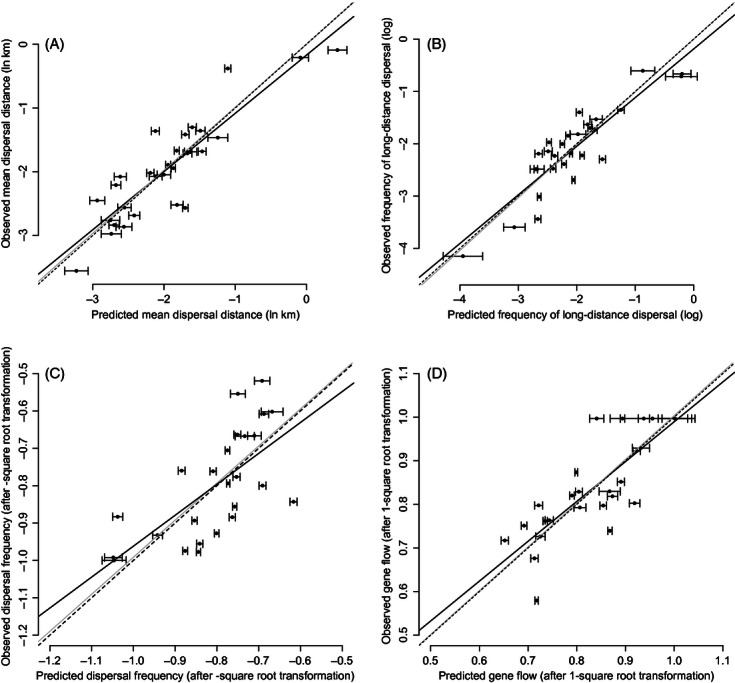
Cross-validations of predictive models for butterfly dispersal: predictions obtained from information on multiple life-history traits, together with wing size (B) or not (panels A, C, D) (see Table 3). A: mean dispersal distance; B: frequency of long-distance dispersal; C: dispersal propensity; D: intensity of gene flow, observed for 25–30 butterfly species, all plotted against the man predicted values and their respective 95% CI (obtained with 100 random partitions). Black lines show the linear regressions; for comparison dotted lines show the slope 1:1, and gray line show the regression forced into 0:0. Stevens *et al*.

Dispersal predicted from the four selected models correctly fitted to the observed measurements (Fig. 1). The predictive ability of these models was much higher than predictions made with wing size only (Table 4). Dispersal in ecological time and gene flow were both well predicted from life-history traits (Table 4, Fig. 1): the slopes of observed versus predicted dispersal ranged between 0.81 and 0.95, to be compared with the generally lower slopes obtained with wing size only (range −5.01 to 0.84), and the predictions obtained were up to five times more precise than those obtained with wing size only, as shown by the inspection of the difference between observations and predictions (Table 4).

**Table 4:** Quality assessment of generalized linear models used to predict dispersal in butterflies. Model description is given in Table 3. Reference level: rightness and precision obtained with a GLM using only wing size

	Rightness			Imprecision		
		
Dispersal measurement	GLM with life-history traits	Reference level	Gain in rightness	GLM with life-history traits	Reference level	Gain in precision
Mean dispersal distance	0.883 ± 0.005	0.819 ± 0.005	+ 0.064***	0.313 ± 0.003	0.636 ± 0.002	× 2.03***
Frequency long-distance dispersal	0.950 ± 0.002	0.788 ± 0.003	+ 0.162***	1.009 ± 0.008	5.265 ± 0.013	× 5.21***
Dispersal propensity	0.809 ± 0.003	0.837 ± 0.003	− 0.027***	0.149 ± 0.0005	0.170 ± 0.0001	× 1.14***
Gene flow	0.889 ± 0.005	−5.015 ± 0.231	+ 5.904***	0.0198 ± 0.0001	0.0311 ± 0.00 003	× 1.57***

Rightness: slope of a regression of observed versus predicte dispersal. Imprecision: average absolute difference between observed and predicted values (for mean dispersal distance and the frequency of long-distance dispersal, given relatively to observed value to account for scale dependency). Mean ± SE over 20 independent bootstraps. Gain in rightness = rightness trait model−reference. Gain in precision = imprecision reference/imprecision trait model. ***P < 0.001 that rightness or imprecision is similar to the reference level; ns: *P* > 0.05.

### Butterfly dispersal

**Figure 2:**
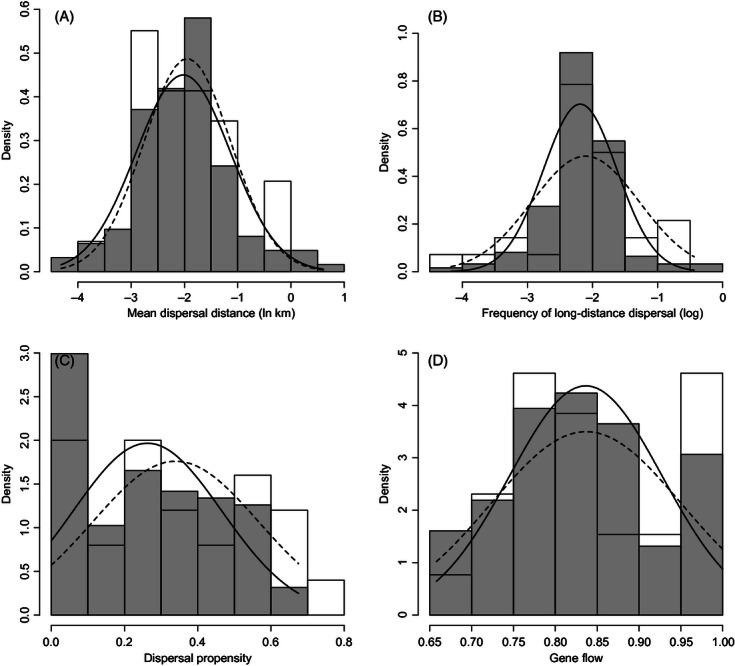
Predicted (dark gray, solid curve) and observed (transparent light gray, dotted curve) density probability and corresponding fitted normal distributions of dispersal ability in butterflies. A: mean dispersal distance; B: probability of long-distance dispersal; C: dispersal propensity; D: gene flow. Observations were direct measurement obtained from mark-recapture surveys (A–C) or indirect estimates obtained via population genetics (D). Predictions were obtained from linear models using wing size and three life-history traits (B) or only information on four life-history traits (A, C, D). Predictions were truncated > 0 for B, and 0–1 for C and D. Predictions are available for *N* = 124–137 species. Stevens *et al*.

We used the four retained models to infer dispersal ability for all NW-European butterflies. The comparison of the observed dispersal measurements to those values inferred from these models showed that the distribution of dispersal ability in predictions and in observations generally converged (Fig. 2; Figs A7–A10 in the online appendix (Data S2)).

Our inferences highlight high dispersal propensity in butterflies: on average about one-third (34%) of the individuals usually leave their natal patch, or the patch of their first capture, and this (observed) proportion reaches 73% in some species (predicted: 78%). A few species however appear much more philopatric: dispersal propensity is less than 5% for the 10% of less dispersive species. Even if they engage often in dispersal, butterflies usually disperse over short distances of only a few hundred meters. The mean dispersal distance is 204 m in predictions (observed = 205 m), and 90% of the species are predicted to have mean dispersal distance ≤ 352 m (observed: < 354 m). A few species are nevertheless observed (and predicted) to move more than an average of 1 km. Very long-distance dispersal however was generally infrequent, and most species are observed (and predicted) to disperse farther than 5 km only rarely: this probability is on average less than 0.01. Here again, a few species appear to have very high dispersal power, with the probability of such long movements reaching 0.25 in some species (observed; higher predictions reached a probability of 0.37 for long-distance dispersal). This high frequency of usually short movements results in quite high levels of gene flow among populations, and the genetic structuring is generally weak: the higher *F*_ST_ observed is 0.177, but 90% of the species have observed *F*_ST_ ≤ 0.078 (in predictions 90% of species have *F*_ST_ ≤ 0.082 and the maximum predicted is 0.117).

## Discussion

### The constituent elements of dispersal are embedded in distinct syndromes

Clearly, as it was predicted from theoretical models (see a review in Ronce and Clobert 2012), dispersal is not evolving independently of other traits, which give rise to predictable syndromes, and consequently a large part of the dispersal variability can be explained by the variation in other phenotypic traits. An interesting result of our study was that different elements of the dispersal process correlated with completely different suites of traits. Although theory remains unclear on this point (Kisdi et al. 2012; Starrfelt and Kokko 2012), some empirical results already suggest that different combinations of life-history traits can be implied at the different steps of the dispersal process (Massot et al. 2002; see also examples in Bonte et al. 2012). The relative roles of phylogenetic inertia, natural selection, sexual selection, or phenotypic plasticity in explaining these relationships among traits certainly deserve further investigation.

The way dispersal is measured in fact reflects different elements of the dispersal process, such as individual movement rates versus gene flow, possibly corresponding to the different definitions of dispersal existing in the literature (Stevens et al. 2010b). The fact that very different suites of traits were retained to predict these various elements of dispersal can be an indication that they are under partially uncoupled selective pressures, which we already suspected from our previous studies (Stevens et al. 2010a,b). Such differences possibly will result in some contrast between the short-term demographic consequences of dispersal and its long-term genetic effects. Alternatively, the fact that dispersal measurements were taken from different samples of species might explain why different syndromes were observed. However, the mean dispersal distance and the frequency of long-distance dispersal were taken on the same species (with the exception of 2 species), and these measurements are associated with different syndromes of life-history traits. Accordingly, the possible artifact due to the use of different species samples does not explain all the differences in the syndromes observed.

This segregation of the dispersal elements in different syndromes of life-history traits might also have deep consequences for the functional diversity of communities facing environmental changes. Indeed, if these correlations have a genetic basis, any selection on a given element of dispersal would have distinct indirect consequences on life-histories, and the dispersal costs at each of the dispersal steps would be paid independently from the costs incurred at other steps (Bonte et al. 2012). Habitat fragmentation for instance was shown to filter species according to their dispersal ability (e.g., Driscoll and Weir 2005; Van Houtan et al. 2007) and to affect the distance moved (Bonte et al. 2010) or the dispersal propensity (Schtickzelle et al. 2006). Our results show that such filtering, or selection, on the mean dispersal distance could entail the functional diversity within butterfly communities, for instance by having indirect effects on the diversity of specialization or of larval growth rate in these communities. However, the process responsible for the observed pattern of trait association is still unknown, and it should be investigated before any proper prediction on the side-effects of the selection acting on dispersal can be made.

In light of our results, dispersal should now be seen as an additional vector in life history, consisting of several uncoupled (or loosely coupled) dispersal elements (dispersal distance, dispersal frequency, gene flow), which increases the array of potential life-history tactics within communities. Accordingly, we must consider each of these dispersal elements as one axis of a species' niche.

### Applications in biodiversity conservation

The advantage of considering several phenotypic traits, and noticeably life-history traits, to infer dispersal ability is considerable. Life history indeed appears to be a very convenient proxy to infer unknown dispersal ability at the species level. For all four dispersal measurements considered here, the inclusion of life-history traits in linear models greatly improved the predictions we would have made from wing size only (Table 4). For three measurements, wing size was not even retained by model selection, and the relative importance of wing size in structuring the variation in the frequency of long-distance dispersal was low (Table 3). These results highlight the fact that this allometry is not efficient in predicting butterfly dispersal, but the existence of dispersal syndromes provides a valuable alternative to make this inference, which in turn is useful for planning actions targeted at preserving biodiversity. Whitmee and Orme (2013) concluded similarly that life-histories offer a convenient opportunity to infer dispersal of mammals. In their study, a wide variety of models that accept very different terms equally well predicted mammal dispersal. In contrast, we showed here that in butterflies, only certain traits that dominated the top-ranked models are really helpful to predict the value of each dispersal element (Table A2).

Measuring movement rates and distances usually requires long and extensive mark-recapture studies or direct tracking, which is always costly and may prove impossible, particularly for rare or endangered species. The trait-based approach developed here proved very useful for inferring mean dispersal distance, dispersal propensity, and even the frequency of long-distance dispersal. Mean dispersal distance is most often needed to feed simulation models, and to help decision making in conservation (Moilanen et al. 2005). For instance, it can be used to infer the spatial grain at which suitable habitats should be distributed in a given landscape to allow a smooth metapopulation functioning (Hanski 1999b; Baguette and Van Dyck 2007; Baguette et al. in press). Long-distance dispersal can also be very crucial for metapopulation persistence, by hampering genetic drift and its negative effects (Lande 1988), or by allowing (re)colonization of distant habitat patches. These maximal movements however are most often ignored in conservation decisions, because they are often not documented. The traits-based model that we developed here to infer the value of this element of dispersal is therefore interesting because it requires measurements of traits that are quite easy to collect from large-scale monitoring, amateurs' reports, or lab rearing, and hence can easily be acquired for many species.

The inference of gene flow by population genetics is also costly as it requires intensive sampling, coupled with laborious and expensive lab work. Therefore, it can be infeasible in some case, especially when conducting multi-species comparisons or if feeding multi-species models is the research goal. The traits-based method derived here from the syndromes associating this element of dispersal to other phenotypic traits offers a reliable alternative to population genetics. Consequently, the relative ability of species to maintain gene flow across space could be inferred for a lot of species, and could be integrated into, for example, conservation plans.

It would be interesting to explore to what extent the trait-based approach allows this inference in taxa other than butterflies. Whitmee and Orme (2013) showed that the trait-based approach reliably predicts natal dispersal distances for mammals: both maximal and median distances were satisfactorily predicted with a variety of trait sets. Life-histories of plants predicted reliably the dispersal mode of seeds (ballistic, wind-assisted, transport by animals, etc.: Thomson et al. 2010). The extent to which life-histories allow predicting seed dispersal distances, seed dispersal frequency, or plant gene flow was however not assessed, probably because their determinants will mainly depend on the dispersal mode the seeds use. In amphibians, we showed that even a poorly informed dataset, with a large amount of missing values, yields accurate predictions of dispersal distances (A. Trochet A, Moulherat S, Calvez O, Schmeller O, Clobert J and Stevens V. M. unpublished). Trait-based methods thus seem promising to infer unknown dispersal ability.

### How can we improve the inferences on dispersal?

Our trait-based approach does offer quick and cheap access to the average dispersal ability of species for which no dispersal data are currently available. This is particularly pertinent in the case of threatened species that may be geographically restricted and for which conservation actions are required but cannot be implemented without considering dispersal. Although even imprecise approximations may strongly improve the power of modeling tools used to predict the fate of populations under changing environmental conditions (Dawson et al. 2011), any solution to refine those predictions is however welcome. We propose here below three ways for such improvements: (i) to go beyond the species level, (ii) to explore other species traits, and (iii) to make use of population patterns that result from dispersal.

Virtually no life-history trait is entirely fixed at the species level, and most are more or less labile, responding quickly to changed environmental conditions, or according to individual conditions (Roff 2002; Clobert et al. 2004). Even discrete traits like voltinism show some plasticity: observations of additional generations in exceptionally hot years are common in butterflies (Bink 1992; Fischer and Fiedler 2002). Dispersal also has substantial variation within species (Schtickzelle et al. 2006; Stevens et al. 2010a). A means of taking this variation into account and making inferences at the infra-species level could be to identify how dispersal varies according to environmental conditions (e.g., climate, habitat quality, fragmentation) and to population characteristics (like density, inbreeding, or kin density) to refine the predictions made at the species level. However, there is currently too little information available to make such generalizations. For those cases that require very precise estimates of dispersal, for instance where dispersal is suspected to evolve locally, like at expanding fronts (Burton et al. 2010), we thus recommend that dispersal should be directly measured or inferred from genetic data collected *in situ* (as suggested by Baguette et al. in press).

Some traits not considered in this study could be used to refine the inferences of dispersal. Palatability of adults for instance is certainly such a trait. Previous studies show that unpalatable species and their mimics have different flight patterns than palatable species (Chai and Srygley 1990), probably because both groups are under contrasting pressures from flying predators. Unfortunately, palatability was not measured for European butterflies, which prevented its integration here.

Finally, factors that are affected by dispersal might also be used to refine the inferences of dispersal, like the geographic range size, or the speed of range expansion. Both relationships are however probably obscured by other processes like vicariance and speciation, habitat suitability, host plant distributions, niche breadth of species along abiotic clines, or evolutionary processes at range margins. For this reason why we did not consider these factors, although their relationships with dispersal distances were shown in birds and in mammals (Sutherland et al. 2000; Bowman et al. 2002; Dawideit et al. 2009).

## Conclusion

The importance of dispersal for the functioning and the evolution of populations cannot be ignored, especially now in times of deep environmental changes. Indeed, this key process determines the response of populations and species to many environmental changes, for instance by limiting local adaptation, or by allowing species to change their distribution (Parmesan 2006; Chen et al. 2011).

We showed here that the constituent elements of dispersal (movement rate, movement distances, and gene flow) form different syndromes of life history, as each is related to a completely different suite of traits. This implies that each of these elements of dispersal should be considered a species life-history trait, and an axis of the species' niche. However, this also means that the changed selective pressures on one or more elements of dispersal might have distinct side-consequences for functional diversity within communities. However, this would be the case only if the observed co-variations among traits are at least partially attributable to genetic co-variation, which is yet to be explored.

An interesting application of these syndromes is the inference of dispersal: the trait-based approach that relies on these syndromes is convenient to infer dispersal ability when data on dispersal are missing. Generally, the lack of reliable dispersal data is considered the most important shortcoming in the use of those simulation models that aim at investigating the extinction risks for populations, at predicting the impact of environmental changes or at assessing the relative effects of alternative mitigation scenarios (e.g., Heikkinen et al. 2006). The trait-based approach we introduce here fills this gap by providing sound inferences of the dispersal abilities for species for which it remains unknown: life-history information indeed is available for nearly three times more butterfly species than is dispersal information. More importantly, this approach allows the explicit consideration of each element of the dispersal process, as well as its association with other phenotypic traits within syndromes of life history. Taking these into account is particularly important if we wish to design efficient conservation plans for preserving the whole array of biodiversity (including for instance genetic diversity or functional diversity) in the face of the combined actions of landscape fragmentation and climate change.

## Data archiving statement

Data used in this study were obtained from previously published studies. A list of the studies is available as Online Supplementary Materials (Data S1).

## Supporting Information

**Data S1:** List of data source for dispersal measurements.

**Data S2:** Supplementary material and supplementary results.

**Table A1.** Summary of 64 generalized linear models (GLM) with each of four dispersal measurements as the response and each of 16 species traits and their quadratic effect proposed as explanatory variables.

**Table A2.** Comparison of the predictive models to others top-ranked models in the selection on GLM. In each case, model 1 is the model used for predictions. R^2^ is unadjusted.

**Figure A1.** Range of trait values in subset of data comprising butterfly species with measured dispersal compared to range observed in 142 butterfly species of N-W Europe. Black: range with 142 species (scaled for reference); green: range in species with measured mean dispersal distances; dark blue: range in species with estimated probability of long-distance dispersal; light blue: range in species with measured dispersal propensity; orange: range in species with estimated gene flow.

**Figure A2.** Illustration of a polynomial effect (dashed black curves = 95% CI) of the flight period in a predictive model for the probability of long distance dispersal in butterflies. Butterflies with known probability of long-distance dispersal have short to medium flight periods (4–15 weeks) while this dispersal measurement should be predicted for species with short to very long flight periods (3–32 weeks). Green lines indicate how we enlarged the range of values used for the predictions to 3–17 weeks, based on the standard deviation of the effect.

**Figure A3.** Illustration of the significant effects of traits and interactions between traits retained to predict the mean dispersal distance in butterflies. Mean dispersal distance is shown on a log km scale. The model is detailed in Table 3 of main text. Effects are shown with 95% CI (dashed curves), except for interaction.

**Figure A4.** Illustration of the significant effects of traits and interactions between traits retained to predict the frequency of long-distance dispersal in butterflies. Frequency of long-distance dispersal is shown on a log scale. The model is detailed in Table 3 of main text. Effects are shown with 95% CI (dashed curves), except for interaction.

**Figure A5.** Illustration of the significant effects of traits and interactions between traits retained to predict the dispersal propensity in butterflies. Dispersal propensity is 1−√proportion of residents. The model is detailed in Table 3 of main text. Effects are shown with 95% CI (dashed curves), except for interactions where CI is not shown.

**Figure A6.** Illustration of the significant effects of traits and interactions between traits retained to predict the intensity of gene flow in butterflies. Gene flow is −√FST. The model is detailed in Table 3 of main text. Effects are shown with 95% CI (dashed curves), except for interactions.

**Figure A7.** Mean dispersal distance predicted from life-history traits and wing size for 138 of the 142 butterfly species of N-W Europe, and 95% CI of the predictions. Details of the model are shown in Table 3 of main text. Red symbols show the observed value for 30 of those species.

**Figure A8.** Mean dispersal distance predicted from life-history traits and wing size for 124 of the 142 butterfly species of N-W Europe, and 95% CI of the predictions. Details of the model are shown in Table 3 of main text. Red symbols show the observed value for 29 of those species.

**Figure A9.** Dispersal propensity predicted from life-history traits for 113 of the 142 butterfly species of N-W Europe, and 95% CI of the predictions. Details of the model are shown in Table 3 of main text. Red symbols show the observed value for 25 of those species.

**Figure A10.** Gene flow predicted from life-history traits for 137 of the 142 butterfly species of N-W Europe, and 95% CI of the predictions. Details of the model are shown in Table 3 of main text. Red symbols show the observed value for 26 of those species.

## References

[b1] Anderson DR, Burnham KP, White GC (1994). AIC model selection in overdispersed capture-recapture data. Ecology.

[b2] Baguette M, Van Dyck H (2007). Landscape connectivity and animal behavior: functional grain as a key determinant for dispersal. Landscape Ecology.

[b3] Baguette M, Petit S, Quéva F (2000). Population spatial structure and migration of three butterfly species within the same habitat network: consequences for conservation. Journal of Applied Ecology.

[b4] Baguette M, Blanchet S, Legrand D, Stevens VM, Turlure C (2013). Individual dispersal, landscape connectivity and ecological networks. Biological Reviews.

[b5] Barton K (2011). http://CRAN.R-project.org/package=MuMIn.

[b6] Beck I, Kitching IJ (2007). Correlates of range size and dispersal ability: a comparative analysis of sphingid moths from the Indo-Australian tropics. Global Ecology and Biogeography.

[b7] Berg MP, Kiers ET, Driessen G, Kooi M, van der Heijden BW, Kuenen F, Liefting M (2010). Adapt or disperse: understanding species persistence in a changing world. Global Change Biology.

[b8] Bink FA (1992). Ecologische Atlas van de Dagvlinders van Noord-West europa.

[b9] Bonte D, Hovestadt T, Poethke HJ (2010). Evolution of dispersal polymorphism and local adaptation of dispersal distance in spatially structured landscapes. Oikos.

[b10] Bonte D, Van Dyck H, Bullock JM, Coulon A, Delgado M, Gibbs M, Lehouck V (2012). Costs of dispersal. Biological Reviews.

[b11] Bowman J, Jaeger JAG, Fahrig L (2002). Dispersal distance of mammals is proportional to home range size. Ecology.

[b12] Burton OJ, Phillips BL, Travis JMJ (2010). Trade-offs and the evolution of life-histories during range expansion. Ecology Letters.

[b13] Chai P, Srygley RB (1990). Predation and the flight, morphology, and temperature of neotropical rain-forest butterflies. American Naturalist.

[b14] Chen IC, Hill JK, Ohlemuller R, Roy DB, Thomas CD (2011). Rapid range shifts of species associated with high levels of climate warming. Science.

[b15] Cizek L, Fric Z, Konvicka M (2006). Host plant defences and voltinism in European butterflies. Ecological Entomology.

[b16] Clobert J, Ims RA, Rousset F, Hanski I, Gaggiotti OE (2004). Causes, mechanims and evolution of dispersal. Ecology, Genetics and Evolution of Metapopulations.

[b17] Clobert J, Le Galliard JF, Cote J, Meylan S, Massot M (2009). Informed dispersal, heterogeneity in animal dispersal syndromes and the dynamics of spatially structured populations. Ecology Letters.

[b18] Clobert J, Baguette M, Benton T, Bullock JM (2012). Dispersal: Ecology and Evolution.

[b20] Dawideit BA, Phillimore AB, Laube I, Leisler B, Bohning-Gaese K (2009). Ecomorphological predictors of natal dispersal distances in birds. Journal of Animal Ecology.

[b21] Dawson TP, Jackson ST, House JI, Prentice IC, Mace GM (2011). Beyond predictions: biodiversity conservation in a changing climate. Science.

[b22] Doebeli M, Dieckmann U (2003). Speciation along environmental gradients. Nature.

[b23] Driscoll DA, Weir T (2005). Beetle responses to habitat fragmentation depend on ecological traits, habitat condition, and remnant size. Conservation Biology.

[b24] Fischer K, Fiedler K (2002). Life-history plasticity in the butterfly *Lycaena hippothoe*: local adaptations and trade-offs. Biological Journal of the Linnean Society.

[b25] Fjerdingstad EJ, Schtickzelle N, Manhes P, Gutierrez A, Clobert J (2007). Evolution of dispersal and life history strategies – *Tetrahymena* ciliates. BMC Evolutionary Biology.

[b26] Franzen M, Nilsson SG (2007). What is the required minimum landscape size for dispersal studies?. Journal of Animal Ecology.

[b27] Fric Z, Klimova M, Konvika M (2006). Mechanical design indicates differences in mobility among butterfly generations. Evolutionary Ecology Research.

[b28] Gittleman JL, Anderson CG, Kot M, Luh HK, Martins EP (1996). Phylogenetic lability and rates of evolution: a comparison of behavioral, morphological and life history traits. Phylogenies and the Comparative Method in Animal Behaviour.

[b29] Hanski I (1998). Metapopulation dynamics. Nature.

[b30] Hanski I (1999a). Metapopulation Ecology.

[b31] Hanski I (1999b). Habitat connectivity, habitat continuity, and metapopulations in dynamic landscapes. Oikos.

[b32] Heikkinen RK, Luoto M, Araujo MB, Virkkala R, Thuiller W, Sykes MT (2006). Methods and uncertainties in bioclimatic envelope modelling under climate change. Progress in Physical Geography.

[b33] Kisdi E, Utz M, Gyllenberg M, Clobert J, Baguette M, Benton T, Bullock JM (2012). Evolution of condition-dependent dispersal. Dispersal Ecology and Evolution.

[b34] Lafranchis T (2000). Les papillons de jour de France, Belgique et Luxembourg et leurs chenilles.

[b35] Lande R (1988). Genetics and demography in biological conservation. Science.

[b36] Li J, Margolies DC (1993). Quantitative genetics of aerial dispersal behaviour and life-history traits in *Tetranychus urticae*. Heredity.

[b37] Lindeman RH, Merenda PF, Gold RZ (1980). Introduction to Bivariate and Multivariate Analysis.

[b38] Martins EP (2000). Adaptation and the comparative method. Trends in Ecology and Evolution.

[b39] Massot M, Clobert J, Lorenzon P, Rossi JM (2002). Condition-dependent dispersal and ontogeny of the dispersal behaviour: an experimental approach. Journal of Animal Ecology.

[b40] Moilanen A, Franco AMA, Early RI, Fox R, Wintle B, Thomas CD (2005). Prioritizing multiple-use landscapes for conservation: methods for large multi-species planning problems. Proceedings of the Royal Society B-Biological Sciences.

[b41] Nathan R (2001). The challenges of studying dispersal. Trends in Ecology and Evolution.

[b42] Ockinger E, Schweiger O, Crist TO, Debinski DM, Krauss J, Kuussaari M, Petersen JD (2010). Life-history traits predict species responses to habitat area and isolation: a cross-continental synthesis. Ecology Letters.

[b43] Parmesan C (2006). Ecological and evolutionary responses to recent climate change. Annual Review of Ecology Evolution and Systematics.

[b44] Peters RH (1983). The Ecological Implications of Body Size.

[b45] Roff DA (2002). Life History Evolution.

[b46] Ronce O (2007). How does it feel to be like a rolling stone? Ten questions about dispersal evolution. Annual Review of Ecology Evolution and Systematics.

[b47] Ronce O, Clobert J, Clobert J, Baguette M, Benton TG, Bullock JM (2012). Dispersal syndromes. Dispersal Ecology and Evolution.

[b48] Schneider C (2003). The influence of spatial scale on quantifying insect dispersal: an analysis of butterfly data. Ecological Entomology.

[b49] Schtickzelle N, Mennechez G, Baguette M (2006). Dispersal depression with habitat fragmentation in the bog fritillary butterfly. Ecology.

[b50] Sekar S (2012). A meta-analysis of the traits affecting dispersal ability in butterflies: can wingspan be used as a proxy?. Journal of Animal Ecology.

[b51] Starrfelt J, Kokko H, Clobert J, Baguette M, Benton TG, Bullock JM (2012). The multicausal nature of dispersal. Dispersal Ecology and Evolution.

[b52] Stevens VM, Pavoine S, Baguette M (2010a). Variation within and between closely related species uncovers high intra-specific variability in dispersal. PLoS ONE.

[b53] Stevens VM, Turlure C, Baguette M (2010b). A meta-analysis of dispersal in butterflies. Biological Reviews.

[b54] Stevens VM, Trochet A, Van Dyck H, Clobert J, Baguette M (2012). How is dispersal integrated in life histories: a quantitative analysis using butterflies. Ecology Letters.

[b55] Sutherland GD, Harestad AS, Price K, Lertzman KP (2000). Scaling of natal dispersal distances in terrestrial birds and mammals. Conservation Ecology.

[b56] Thomson FJ, Moles AT, Auld TD, Ramp D, Ren SQ, Kingsford RT (2010). Chasing the unknown: predicting seed dispersal mechanisms from plant traits. Journal of Ecology.

[b57] Turlure C, Schtickzelle N, Baguette M (2010). Resource grain scales mobility and adult morphology in butterflies. Landscape Ecology.

[b58] Van Houtan KS, Pimm SL, Halley JM, Bierregaard RO, Lovejoy TE (2007). Dispersal of Amazonian birds in continuous and fragmented forest. Ecology Letters.

[b59] Whitmee S, Orme CDL (2013). Predicting dispersal distance in mammals: a trait based approach. Journal of Animal Ecology.

