# Supplementary material for: Dispersal syndromes and the use of life-histories to predict dispersal
Source: Evol Appl. 2013 Feb 11;6(4):630–42. doi: 10.1111/eva.12049 (PMC3684744; doi:10.1111/eva.12049)
Supplement: Supplementary file 2 [file eva0006-0630-SD2.pdf]

Supplementary material for ñDispersal syndromes and the use of life-histories to predict dispersalò by Stevens VM, Trochet A, Blanchet S, Moulherat S, Clobert J & Baguette M

1. Identification of quadratic relationships between dispersal and species traits

A preliminary analysis of the relationships between dispersal measurements and butterfly traits allowed to identify putatively non-linear relationships between traits. When the effect of the quadratic term of a trait was even marginally significant in a GLM (i.e. with $P < 0.10$), the trait effect was modeled with a polynomial effect of degree 2 in the model construction for predictive models. Table A1 summarizes this preliminary analysis.

Table A1. Summary of 64 generalized linear models (GLM) with each of four dispersal measurements as the response and each of 16 species traits and their quadratic effect proposed as explanatory variables.

		Mean dispersal distance			Frequency of long-distance dispersal			Dispersal propensity			Gene flow		
Independent variable	Model parameters	Est.	SE	P>|t|	Est.	SE	P>|t|	Est.	SE	P>|t|	Est.	SE	P>|t|
Fecundity	Intercept	-2.40	0.55	<0.01	-2.48	0.63	<0.01	-0.72	0.15	<0.01	0.67	0.08	<0.01
	Fecundity	-0.04	0.26	0.87	-0.03	0.29	0.91	-0.08	0.08	0.38	0.04	0.04	0.31
	Fecundity²	0.03	0.02	0.23	0.03	0.03	0.39	0.01	0.01	0.26	0.00	0.00	0.93
Adult lifetime	Intercept	-3.77	1.39	<0.05	-4.11	1.39	<0.01	-1.05	0.29	<0.01	0.73	0.13	<0.01
	Adult lifetime	0.19	0.15	0.23	0.24	0.15	0.12	0.02	0.03	0.44	0.00	0.01	0.68
	Adult lifetime²	-0.00	0.00	0.28	**-0.01**	**0.00**	**0.09**	-0.00	0.00	0.50	0.00	0.00	0.89
Voltinism	Intercept	-0.14	1.37	0.92	-1.63	1.49	0.29	-0.12	0.45	0.80	0.75	0.29	<0.05
	Voltinism	-1.48	0.91	0.11	-0.62	0.99	0.54	-0.43	0.32	0.19	0.02	0.20	0.93
	Voltinism²	**0.25**	**0.12**	**0.05**	0.14	0.14	0.32	0.05	0.05	0.28	0.00	0.03	0.89
Larval growth rate	Intercept	-0.72	0.51	0.17	-0.61	0.50	0.23	-0.63	0.21	<0.01	0.95	0.10	<0.01
	Larval growth rate	-0.03	0.01	<0.05	-0.03	0.01	<0.05	-0.00	0.01	0.43	0.00	0.00	0.32
	Larval growth rate²	-0.00	0.00	0.14	**0.00**	**0.00**	**0.09**	0.00	0.00	0.43	0.00	0.00	0.40
Ripe egg load	Intercept	-2.75	0.70	<0.01	-2.84	0.71	<0.01	-0.79	0.11	<0.01	0.84	0.09	<0.01

	Ripe egg load	0.33	0.32	0.31	0.37	0.33	0.27	-0.02	0.06	0.67	-0.03	0.04	0.50
	Ripe egg load²	-0.03	0.03	0.41	-0.04	0.03	0.28	0.00	0.01	0.51	0.01	0.00	0.18
Ovigeny index	Intercept	-1.44	0.21	<0.01	-1.94	0.25	<0.01	-0.78	0.05	<0.01	0.90	0.03	<0.01
	Ovigeny index	-4.21	1.43	<0.01	-2.60	1.78	0.16	-0.07	0.42	0.86	-0.47	0.27	0.10
	Ovigeny index²	**3.56**	**1.38**	**<0.05**	2.26	1.71	0.15	0.03	0.39	0.94	0.34	0.27	0.21
Female maturation	Intercept	-1.13	0.82	0.18	-1.72	0.75	<0.05	-0.55	0.14	<0.01	0.63	0.10	<0.01
	Female maturation	-0.68	0.56	0.23	-0.08	0.52	0.88	-0.20	0.09	<0.05	0.10	0.07	0.13
	Female maturation²	0.12	0.08	0.18	-0.02	0.08	0.81	**0.03**	**0.01**	**<0.05**	-0.01	0.01	0.32
Overwintering stage	Intercept	-1.82	0.53	<0.01	-2.32	0.56	<0.01	-0.71	0.19	<0.01	0.92	0.09	<0.01
	Overwintering stage	-0.28	0.29	0.34	-0.06	0.30	0.84	-0.05	0.21	0.82	-0.08	0.06	0.22
	Overwintering stage²	**0.06**	**0.03**	**0.07**	0.04	0.04	0.33	0.00	0.05	0.95	0.02	0.01	0.13
Flight period	Intercept	-4.30	1.08	<0.01	-5.66	0.85	<0.01	-0.98	0.23	<0.01	0.69	0.07	<0.01
	Flight period	0.58	0.27	<0.05	0.97	0.21	<0.01	0.04	0.06	0.49	0.02	0.01	0.08
	Flight period²	**-0.03**	**0.01**	**<0.05**	**-0.06**	**0.01**	**<0.01**	-0.00	0.00	0.56	-0.00	0.00	0.23
Thermal tolerance	Intercept	-3.14	0.79	<0.01	-2.52	0.84	<0.01	-1.07	0.12	<0.01	0.59	0.16	<0.01
	Thermal tolerance	0.51	0. 33	0.13	0.21	0.35	0.55	0.15	0.05	<0.01	0.07	0.06	0.26
	Thermal tolerance²	-0.04	0.03	0.15	-0.02	0.03	0.52	**-0.01**	**0.00**	**<0.01**	-0.00	0.01	0.47
Adult habitat range	Intercept	-0.51	0.70	0.47	-1.46	0.81	0.08	-0.64	0.16	<0.01	0.67	0.07	<0.01
	Adult habitat range	-1.75	0.66	<0.05	-0.93	0.75	0.23	-0.17	0.15	0.27	0.08	0.04	0.10

	Adult habitat range²	**0.39**	**0.13**	**<0.01**	0.23	0.15	0.15	0.04	0.03	0.25	-0.00	0.01	0.50
Larval dietary breadth	Intercept	-3.35	1.64	0.05	-1.83	1.68	0.28	-1.27	0.22	<0.01	0.90	0.15	<0.01
	Larval dietary breadth	0.97	1.21	0.43	-0.03	1.22	0.98	0.39	0.18	<0.05	-0.05	0.12	0.71
	Larval dietary breadth²	-0.16	0.21	0.47	-0.02	0.21	0.94	**-0.07**	**0.03**	**<0.05**	0.01	0.02	0.73
Myrmecophily	Intercept	-1.90	0.17	<0.01	-2.12	0.18	<0.01	-0.78	0.03	<0.01	0.83	0.02	<0.01
	Myrmecophily	-0.28	0.43	0.52	0.09	0.27	0.74	0.09	0.09	0.34	-0.06	0.05	0.25
	Myrmecophily²	0.05	0.10	0.60	-0.01	0.03	0.70	-0.02	0.01	0.21	0.01	0.01	0.18
Laying precision	Intercept	-2.41	0.72	<0.01	-2.28	0.73	<0.01	-1.05	0.20	<0.01	0.76	0.13	<0.01
	Laying precision	0.08	0.33	0.81	-0.06	0.34	0.86	0.19	0.12	0.12	0.02	0.06	0.69
	Laying precision²	0.00	0.04	0.92	0.02	0.04	0.62	**-0.03**	**0.02**	**0.10**	-0.00	0.01	0.83
Mate location	Intercept	-2.72	0.95	<0.01	-2.03	0.88	<0.05	-0.94	0.20	<0.01	0.79	0.16	<0.01
	Mate location	0.87	0.63	0.18	0.28	0.62	0.66	0.12	0.13	0.39	0.02	0.11	0.85
	Mate location²	**-0.18**	**0.10**	**0.08**	-0.09	0.10	0.39	-0.02	0.02	0.35	-0.00	0.02	0.92
Wing size	Intercept	-5.18	1.41	<0.01	-13.5	14.06	0.35	-1.69	2.94	0.57	-0.38	1.88	0.84
	Log(wing size)	0.24	0.12	0.07	5.99	9.29	0.52	0.29	0.92	0.88	0.80	1.28	0.54
	Log(wing size)²	-0.00	0.00	0.18	-0.72	0.52	0.64	0.00	0.31	0.99	-0.13	0.22	0.55

Bold cells show which effects should be modeled with polynomial effects in models aiming at predicting dispersal in butterflies (see main text).

2. Limits to predictions

Predictions from GLM might be problematic in case of skewed range of trait values in the informed dataset (i.e. the species with dispersal measurement available) relatively to the dataset to which the model will be applied. To identify such flaw, we compared the range of values for each trait to the range observed in the whole dataset (Figure A1). This comparison showed that the four informed datasets were skewed for a variety of traits. When traits with skewed range were retained by model selection, we restricted our predictions to butterflies with trait values within the range observed in the dataset from which the predictive model was built (i.e. in species with measured dispersal), as depicted in Fig. A2.

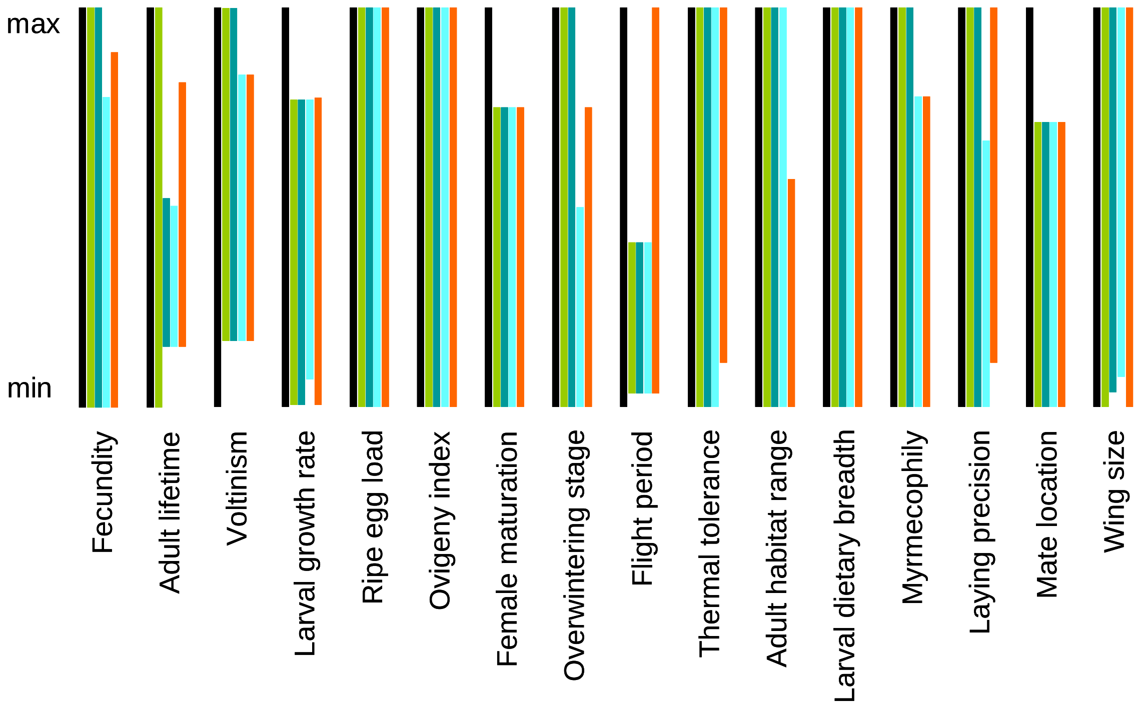

Figure A1. Range of trait values in subset of data comprising butterfly species with measured dispersal compared to range observed in 142 butterfly species of N-W Europe. Black: range with 142 species (scaled for reference); green: range in species with measured mean dispersal distances; dark blue: range in species with estimated probability of long-distance dispersal; light blue: range in species with measured dispersal propensity; orange: range in species with estimated gene flow.

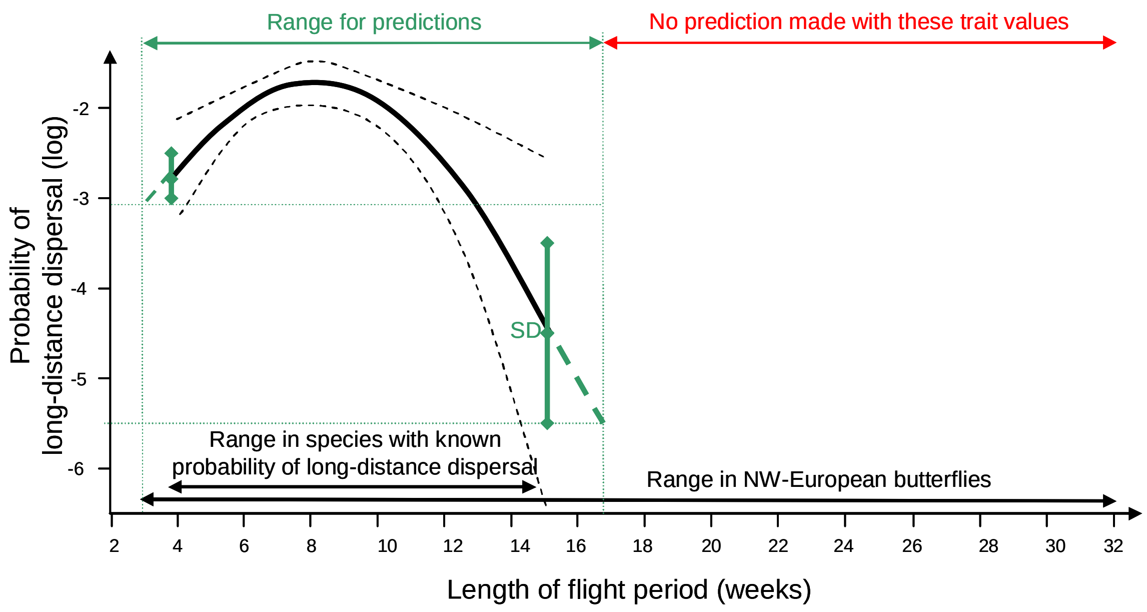

Figure A2. Illustration of a polynomial effect (dashed black curves = 95% CI) of the flight period in a predictive model for the probability of long distance dispersal in butterflies. Butterflies with known probability of long-distance dispersal have short to medium flight periods (4-15 weeks) while this dispersal measurement should be predicted for species with short to very long flight periods (3-32 weeks). Green lines indicate how we enlarged the range of values used for the predictions to 3-17 weeks, based on the standard deviation of the effect.

3. Trait models

We selected one single model to predict each dispersal measurement. Seven species traits and some interactions among them were used in those predictive GLM. The top-ranked models (i.e. within 2 points of AIC from the very best model) usually accept the same terms as the one chosen for predictions, as shown in Table A2. Figs A3-A6 show the effect of each trait retained in these models.

Table A2. Comparison of the predictive models to others top-ranked models in the selection on GLM. In each case, model 1 is the model used for predictions. R^2 is unadjusted.

Dispersal	Model # (ΔAIC<2)	R^2	Number of traits in common with the model finally retained	Number of other traits
Dispersal propensity	1	0.62	4/4	0
	2	0.59	3/4	1
	3	0.59	3/4	1
	4	0.59	3/4	1
	5	0.59	3/4	1
	6	0.53	3/4	0
Mean dispersal distance	1	0.86	7/7	0
	2	0.82	7/7	0
Frequency of long-distance dispersal	1	0.90	6/6	0
	2	0.88	5/6	0
	3	0.88	5/6	0
	4	0.88	4/6	1
	5	0.89	5/6	1
	6	0.81	3/6	0
	7	0.83	4/6	0
	8	0.85	4/6	0
	9	0.87	4/6	0
Gene flow	1	0.77	4/4	0

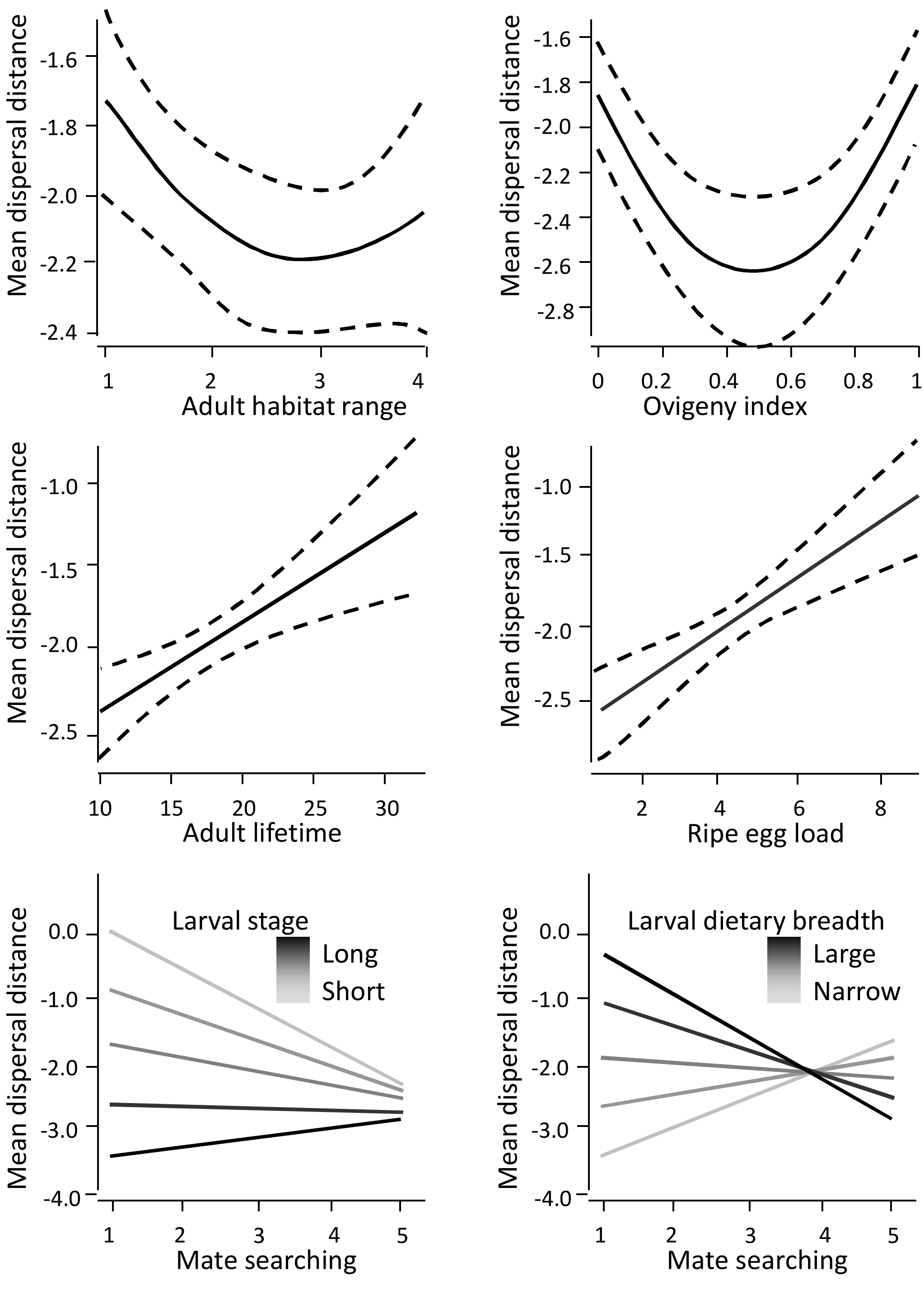

Figure A3. Illustration of the significant effects of traits and interactions between traits retained to predict the mean dispersal distance in butterflies. Mean dispersal distance is shown on a ln km scale. The model is detailed in Table 3 of main text. Effects are shown with 95%CI (dashed curves), except for interaction.

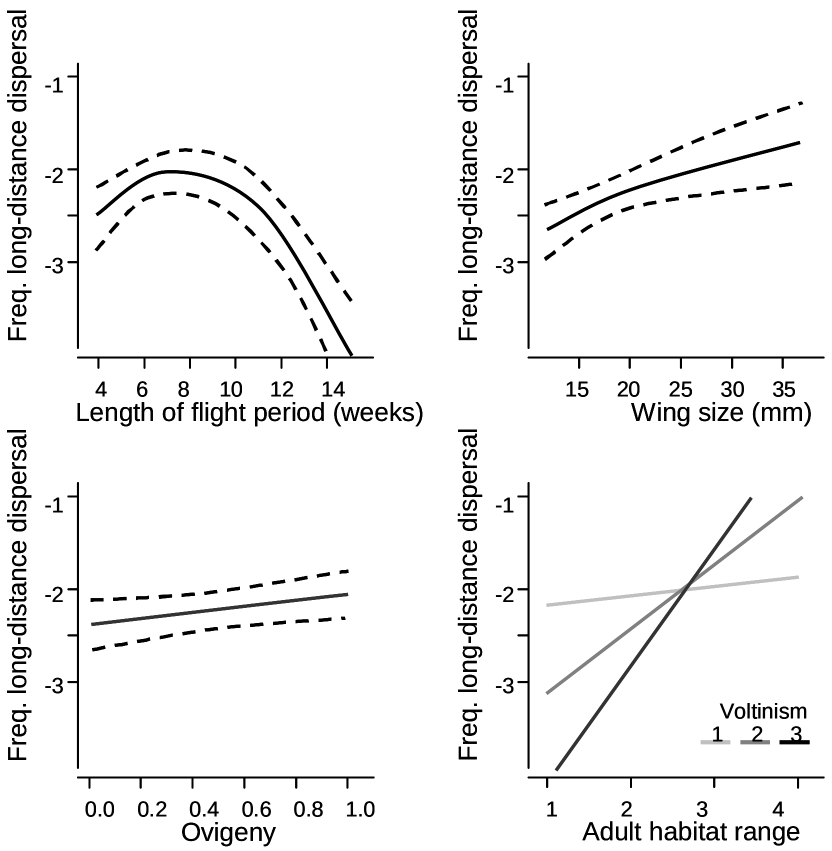

<u>Figure A4</u>. Illustration of the significant effects of traits and interactions between traits retained to predict the frequency of long-distance dispersal in butterflies. Frequency of long-distance dispersal is shown on a log scale. The model is detailed in Table 3 of main text. Effects are shown with 95%CI (dashed curves), except for interaction.

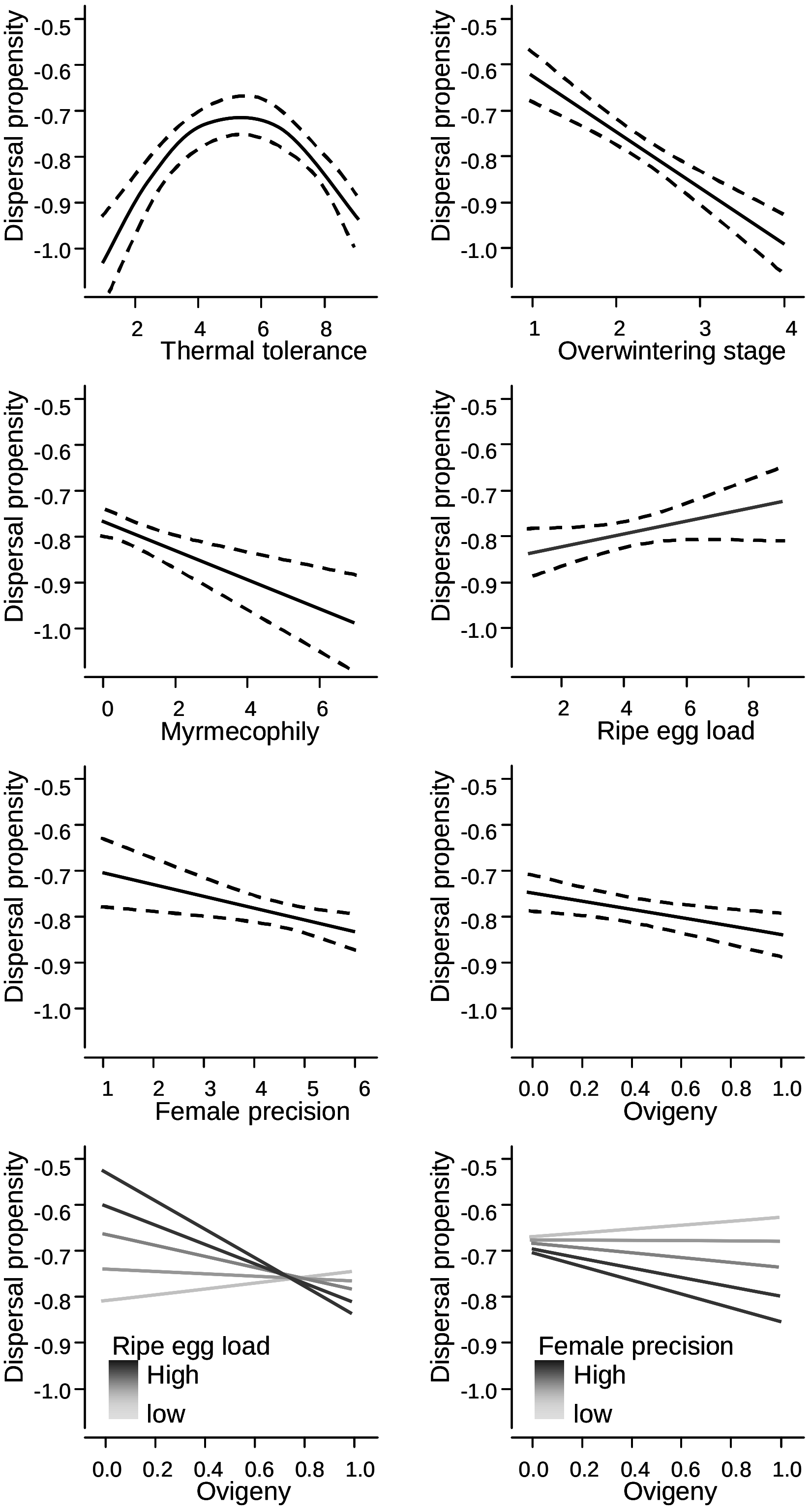

<u>Figure A5.</u> Illustration of the significant effects of traits and interactions between traits retained to predict the dispersal propensity in butterflies. Dispersal propensity is 1-ã proportion of residents. The model is detailed in Table 3 of main text. Effects are shown with 95%CI (dashed curves), except for interactions where CI is not shown.

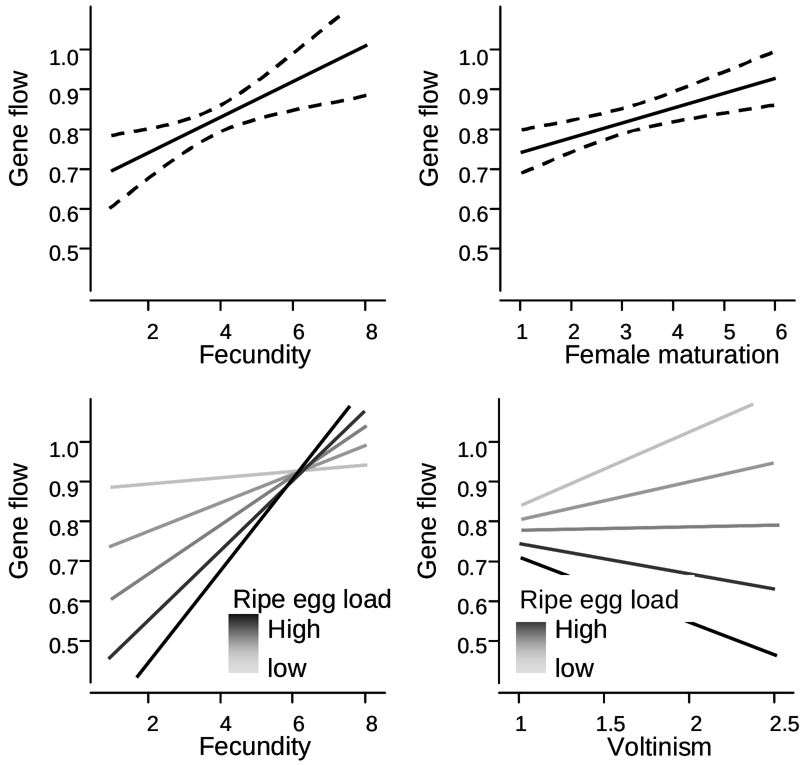

Figure A6. Illustration of the significant effects of traits and interactions between traits retained to predict the intensity of gene flow in butterflies. Gene flow is ï ãF_{ST}. The model is detailed in Table 3 of main text. Effects are shown with 95%CI (dashed curves), except for interactions.

4. Inferences for butterfly dispersal from life-history traits

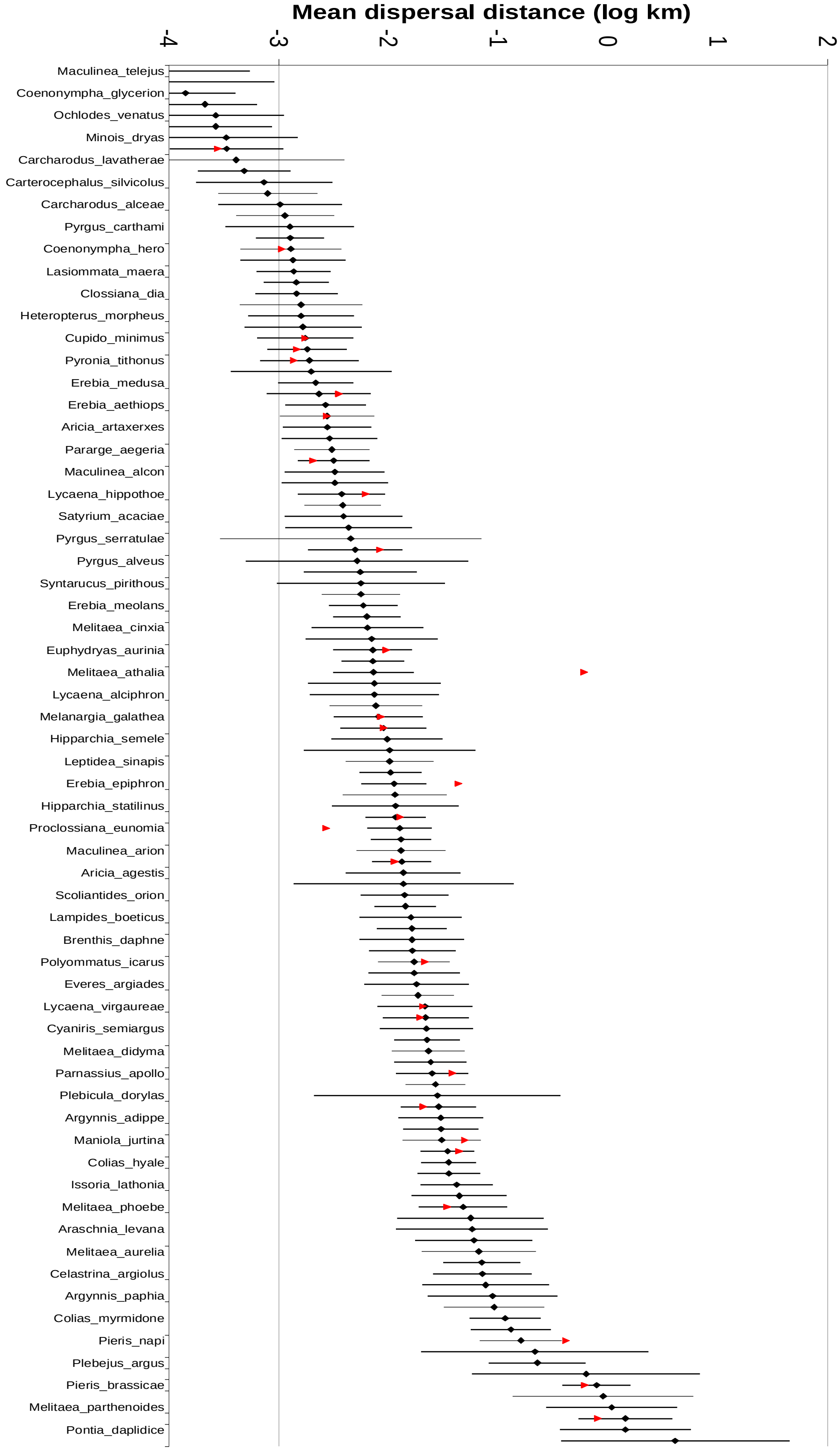

Figure A7. Mean dispersal distance predicted from life-history traits and wing size for 124 of the 142 butterfly species of N-W Europe, and 95%CI of the predictions. Details of the model are shown in Table 3 of main text. Red symbols show the observed value for 29 of those species.

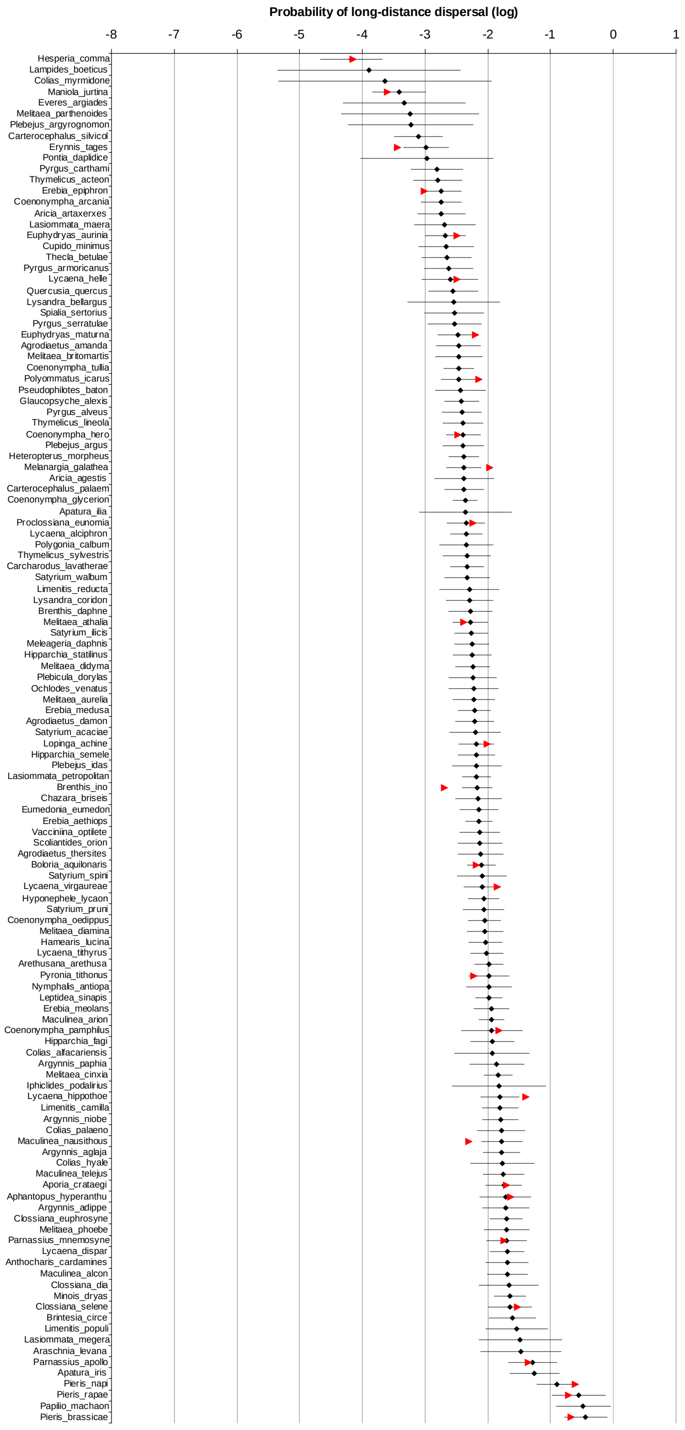

Figure A8. Probability of long-distance dispersal predicted from life-history traits and wing size for 124 of the 142 butterfly species of N-W Europe, and 95%CI of the predictions. Details of the model are shown in Table 3 of main text. Red symbols show the observed value for 28 of those species.

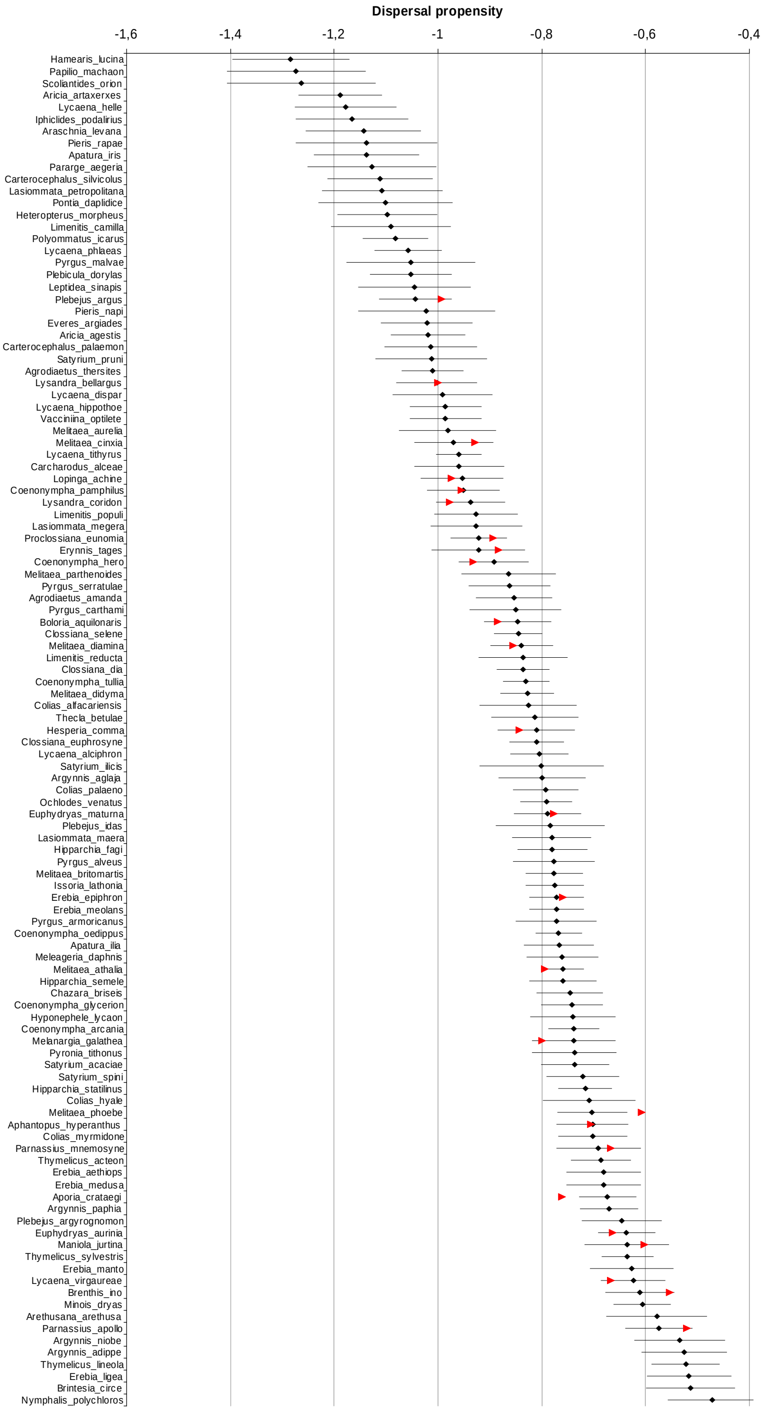

Figure A9. Dispersal propensity predicted from life-history traits for 113 of the 142 butterfly species of N-W Europe, and 95%CI of the predictions. Details of the model are shown in Table 3 of main text. Red symbols show the observed value for 25 of those species.

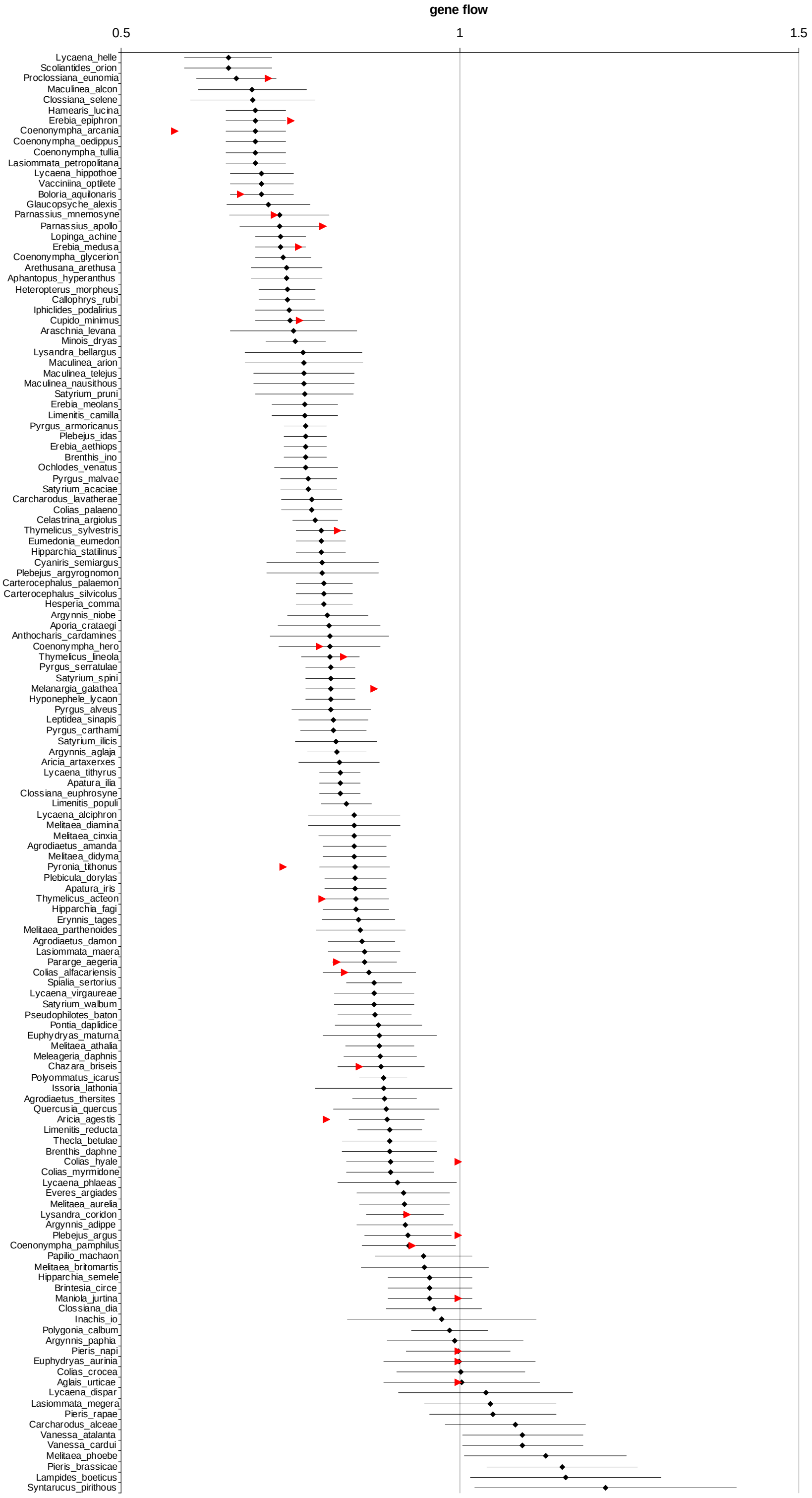

Figure A10. Gene flow predicted from life-history traits for 137 of the 142 butterfly species of N-W Europe, and 95%CI of the predictions. Details of the model are shown in Table 3 of main text. Red symbols show the observed value for 26 of those species.
